# Integrated ovarian mRNA and miRNA transcriptome profiling characterizes the genetic basis of prolificacy traits in sheep (*Ovis aries*)

**DOI:** 10.1186/s12864-017-4400-4

**Published:** 2018-01-29

**Authors:** Kisun Pokharel, Jaana Peippo, Mervi Honkatukia, Arja Seppälä, Johanna Rautiainen, Nasser Ghanem, Tuula-Marjatta Hamama, Mark A. Crowe, Magnus Andersson, Meng-Hua Li, Juha Kantanen

**Affiliations:** 1 Green Technology, Natural Resources Institute of Finland (Luke), Myllytie 1, Jokioinen, Finland; 2 Eastman Chemical Company, Tammasaarenkatu 1, Helsinki, Finland; 3 Lammasmaailma Oy, Ylöjärvi, Finland; 4 Department of Animal Production, Faculty of Agriculture, Cairo University, Giza, Egypt; 5 School of Veterinary Medicine, University College Dublin, Belfield, Dublin 4, Ireland; 6 Department of Production Animal Medicine, University of Helsinki, Helsinki, Finland; 7 CAS Key Laboratory of Animal Ecology and Conservation Biology, Institute of Zoology, Chinese Academy of Sciences (CAS), Beijing, China

**Keywords:** Finnsheep, Texel, F1 cross, Flushing diet, Ovulation rate, *GDF9*

## Abstract

**Background:**

The highly prolific breeds of domestic sheep (*Ovis aries*) are globally valuable genetic resources for sheep industry. Genetic, nutritional and other environmental factors affect prolificacy traits in sheep. To improve our knowledge of the sheep prolificacy traits, we conducted mRNA-miRNA integrated profiling of ovarian tissues from two pure breeds with large (Finnsheep) vs. small (Texel) litter sizes and their F1 crosses, half of which were fed a flushing diet.

**Results:**

Among the samples, 16,402 genes (60.6% known ovine genes) were expressed, 79 novel miRNAs were found, and a cluster of miRNAs on chromosome 18 was detected. The majority of the differentially expressed genes between breeds were upregulated in the Texel with low prolificacy, owing to the flushing diet effect, whereas a similar pattern was not detected in the Finnsheep. F1 ewes responded similarly to Finnsheep rather than displaying a performance intermediate between the two pure breeds.

**Conclusions:**

The identification and characterization of differentially expressed genes and miRNAs in the ovaries of sheep provided insights into genetic and environmental factors affecting prolificacy traits. The three genes (*CST6*, *MEPE* and *HBB*) that were differentially expressed between the group of Finnsheep and Texel ewes kept in normal diet appeared to be candidate genes of prolificacy traits and will require further validation.

**Electronic supplementary material:**

The online version of this article (10.1186/s12864-017-4400-4) contains supplementary material, which is available to authorized users.

## Background

In domestic sheep (*Ovis aries*), a large range of litter sizes (number of offspring from 1 to 9) has been observed among and within breeds, in contrast to several other domestic animal species in which females generally have either 1–2 (cattle, horses and goats) or more than 3 offspring (e.g., dogs and pigs). Highly prolific breeds of domestic sheep are valuable genetic resources for the global sheep industry. Two important fertility traits, the ovulation rate and litter size, are of high economic value in sheep [1]. For thousands of years, animals with good fertility have been favoured in the domestication process. Modern animal breeding and quantitative genetic studies have shown that traditional selective breeding for traits has been slow, with low genetic gains [2–4]. The low heritability and high individual variation of fertility traits have been crucial limitations to improving the reproductive performance of breeds, and typically the traits have been improved through crossbreeding with a prolific breed [1]. These limitations of traditional selection methods, which are mainly based on phenotypic characteristics, have led to a growing interest in the identification and characterization of genes and genomic regions that regulate these economically important traits.

During reproductive processes, ovulation and follicle numbers are regulated by the hypothalamic-pituitary-gonadal axis (HPG-axis) and involve sequential waves of endocrine events, including changes in progesterone, follicle-stimulating hormone (FSH), and luteinizing hormone (LH) levels [5]. Nutrition is one of the most important environmental factors affecting reproductive performance in sheep. Flushing, the practice of increasing nutrient intake and body condition prior to and during breeding, has been shown to affect the ovulation rate and, therefore, the lambing rate [6–9]. However, the genetics and physiological mechanisms that underlie this effect have not been well elucidated. Therefore, an in-depth understanding of how nutrition affects folliculogenesis and the ovulation rate is essential for facilitating targeted nutrition and improving overall sheep fertility [7].

Earlier studies have indicated that fertility traits in sheep can be regulated by individual genetic markers with major effects, which are known as fecundity genes [10, 11], or by polygenic effects, particularly in prolific breeds, such as Finnsheep and Romanov [12]. A number of mutations in major functional genes, such as *GDF9*, *BMP15*, *BMPR1B* and *B4GALNT2*, control ovulation rate and litter size in sheep [10, 13–16]. Recently, transcriptomic analyses via RNA-seq and microarray analysis of different sheep reproductive organs have provided further insights into the gene expression landscapes of these tissues, and a few novel genes (e.g., *PTGS2*, *STAR*, *UCP2*, *IL1A* and *IL1B*) have been found to be associated with prolificacy in sheep [17–20]. However, none of the earlier studies have involved cross-bred ewes, an excellent model for understanding the heritability of important genes.

In the present study, we applied modern genomic methods to identify and characterize genetic markers, candidate genes and pathways associated with reproductive traits. The selection of tissue samples and the underlying computational methods used were based on our pilot studies [21, 22]. We sequenced both the mRNA and miRNA from biopsied ovarian tissues from two sheep breeds, Finnsheep and Texel, and their F1 crosses. Finnsheep, a prolific native breed, have an average litter size of 2.7 [23, 24], whereas Texel sheep have an average litter size of 1.5 [25]. Finnsheep have been used in crossbreeding with local breeds in many counties for decades to improve the prolificacy of these local breeds. The F1 cross was included into the study to investigate the transmission of the prolificacy phenotype into F1 generation. A total of 39 mRNA libraries representing 31 ewes were sequenced. An integrated analytic approach combining mRNA and miRNA sequencing data was used to investigate the gene expression patterns in the sheep ovaries. The effects of diet on the global ovarian mRNA and miRNA profiles was also investigated by maintaining approximately half of the ewes from each breed group on a flushing diet and the other half on a normal diet. The gene expression profiles of pure breeds were compared by using the subset of control diet ewes. Network analysis of the differentially expressed genes and miRNAs was also implemented to reveal the gene ontology terms and pathways associated with developmental changes and reproduction in sheep. This study elucidates the gene expression landscape in the ovaries and the factors affecting reproductive efficiency in sheep.

## Results and discussion

### Pedigree verification, feeding experiments and phenotypic observations

The aim of the present study was to investigate possible differences in the ovarian transcriptomes of two breeds and their F1 crosses. The animals were selected on the basis of their pedigree records. The single-nucleotide polymorphism (SNP)-based results for the genetic relationships between individuals confirmed the ancestries of the groups derived prior to the feeding experiment on the basis of the pedigree records. The multidimensional scaling (MDS) plot based on IBS scores from SNP genotype data indicated three clearly different, non-overlapping clusters (Additional file 1: Fig. S1A), and F1 cross individuals were located between Finnsheep and Texel.

Ewes in the experiment were mature, with an average age and weight of 4.25 years and 71.2 kg, respectively (Additional file 1: Table S1). On average, the Finnsheep ewes had a larger litter size of 2.7, and Texel had the lower litter size of 1.8, whereas their F1 crosses had litter sizes intermediate between those of the two breeds, with an average litter size of 2.4. Most of the ewes were in good body condition at the start of the flushing period, with an average body condition score (BCS) of 3.0. Any potential confounding effects of age, weight or BCS at the start of the trial were eliminated by allocating the animals between the two treatment groups (see Additional file 1: Tables S2 and S3). The feeds used in the experiment consisted of hay, minerals, oats and rapeseed meal (Additional file 1: Table S2). A statistically significant flushing effect was detected for BCS (Additional file 1: Table S3), although the average rise in BCS was only 0.26 within six weeks. This increase was small, given the expected rise of 1 BCS unit within six weeks in thin animals grazing on good pasture [26]. The BCS of the animals that did not receive the flushing diet remained constant during the trial. The low levels of non-esterified fatty acids (NEFA; <0.4 mmol/l) and beta-hydroxybutyrate (β-HB; <0.5 mmol/l) indicated that the animals were not mobilizing body reserves during the trial. Flushing elevated blood urea concentrations in the flushing group (5.25 mmol/l) compared with the non-flushing group (4.37 mmol/l), most probably because of the rapeseed meal included in the flushing diet (Additional file 2).

### mRNA expression in ovaries

The overall quality of the mRNA-seq reads was very good, with average quality scores > 28. After removal of the low-quality reads and universal Illumina adapters present in approximately 5% of the data, an average of 114.5 million reads per sample (Additional file 1: Table S4) were used for further analyses. The percentage of reads from each sample that mapped to the ovine reference genome ranged from 64.7% to 84.5%.

**Fig. 1:**
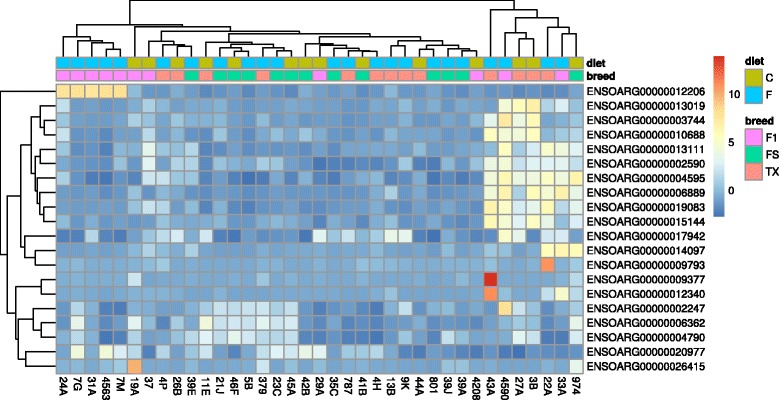
Heatmap plot of the top 20 genes with the highest genetic variance across all samples. Respective diet condition (C = control diet; F = flushing diet) and breed groups (FS = Finnsheep; TX = Texel; F1 = F1 cross of Finnsheep and Texel) of each samples in x-axis are presented at the top of the heatmap

Altogether, 16,402 genes (baseMean ≥5) were expressed in the samples, which represented 60.6% of the known (27,054) ovine genes. Moreover, breed-specific expression revealed that the largest number of genes were expressed in F1 crosses (*n* = 17,345), followed by Finnsheep (*n* = 16,079) and Texel (*n* = 16,039). Unlike SNP-based grouping (Additional file 1: Fig. S1A), initial clustering of highly expressed genes from all samples did not reveal breed- or diet-specific groups (Additional file 1: Fig. S1B), thus suggesting little gene expression variance within and between groups. This conclusion was further illustrated by the observation that among the top 500 most expressed genes, 446 were common to all three groups (Additional file 1: Fig. S2). The top 20 Ensembl IDs with the highest variance across samples included known sheep genes, such as *CA5A* (*ENSOARG000000012206*), *TRH* (*ENSOARG00000001309*), *TFF2* (*ENSOARG000000010688*), *IL1RL1* (*ENSOARG00000013111*), *SPP1* (*ENSOARG0000002590*), *OXT* (*ENSOARG00000004595*), *KIAA1324* (*ENSOARG0000009083*) and *SERPINA5* (*ENSOARG00000015144*) (Fig. 1, Table 1). As shown in the Venn diagram, cross-bred ewes were genetically closer to Finnsheep (25 shared genes) than Texel (15 shared genes). However, only 12 of the 500 top expressed genes were shared by Finnsheep and Texel ewes. We believe the more similar behaviour of F1 ewes to Finnsheep may be associated with epigenetic inheritance of both paternally and maternally expressed imprinted genes in the ovaries. Moreover, because the ovary biopsies including follicular and connective tissues were used for RNA extraction, the heterogeneity of the tissue itself might have affected the overall gene expression dynamics. Thus, future experiments using methods such as manual microdissection [27] may provide a clearer picture of the different cell types.

**Table 1:** List of the top 20 genes with highest variance across samples. Four of these genes were also differentially expressed between one or more conditions and their expression differences are recorded in “Additional files (AF)” column

Ensembl Gene ID	Count	Gene name	Description	Chromosome	Additional files (AF)
ENSOARG00000012206	1081.7	CA5A	Carbonic anhydrase VA, mitochondrial	14	AF3, AF4, AF5
ENSOARG00000013019	151.4	TRH	Thyrotropin-releasing hormone	19	AF4, AF5
ENSOARG00000003744	113.5	HP*	Haptoglobin	14	
ENSOARG00000010688	769.3	TFF2	Trefoil factor 2	1	
ENSOARG00000013111	2415.6	IL1RL1	Interleukin 1 receptor like 1	3	
ENSOARG00000002590	6270.5	SPP1	Osteopontin precursor	6	
ENSOARG00000004595	9875.9	OXT	oxytocin/neurophysin I prepropeptide	13	
ENSOARG00000006889	148.7	SERPINB2*	Serpin family B member 2	23	
ENSOARG00000019083	790.2	KIAA1324	KIAA1324	13	AF5
ENSOARG00000015144	963.6	SERPINA5	Serpin peptidase inhibitor, clade A (alpha-1 antiproteinase, antitrypsin), member 5	18	AF5
ENSOARG00000017942	1108.1	CHGB	Chromogrannin B	13	
ENSOARG00000014097	16.6	CPNE7	copine 7	14	
ENSOARG00000009793	2.7	AHSP	alpha hemoglobin stabilizing protein	24	
ENSOARG00000009377	3.2	UGT*	UDP-glucuronosyltransferase	6	
ENSOARG00000012340	2.8	C1QL2	complement component 1, q subcomponent-like 2	2	
ENSOARG00000002247	247.9			19	
ENSOARG00000006362	502.2	GCG	Glucagon	2	
ENSOARG00000004790	714.8	MMP13	Matrix metallopeptidase 13	15	
ENSOARG00000020977	2677.4	CYP19A1	aromatase	7	
ENSOARG00000026415	23.9			25	

Genes encoding prostaglandins, NADH dehydrogenases and cytochromes were among the most highly expressed genes detected in the sheep ovaries. The top ten most expressed genes were *ND1*, *ND2*, *ND4*, *ND5*, *COX1*, *COX2*, *COIII*, *CYTB*, *ATP6* and *ENSOARG00000006149*, which represented approximately 12% of the total clean reads. Interestingly, the top nine genes expressed in ovarian samples belonged to the mitochondrial genome, and a BLAST search against the non-redundant (NR) database showed that the protein sequence of *ENSOARG00000006149* was 100% identical to elongation factor 1-alpha 1 (*EEF1A1*) from mammals (including human, mouse and cattle). The sheep mitochondrial genome (16,616 bps) consists of 13 coding genes and 24 non-coding genes. All 13 coding genes were expressed together with 9 non-coding genes (2 rRNAs and 7 tRNAs). Moreover, the expressed mitochondrial transcriptome represented approximately 14% (total base mean: 4,380,733) of the expressed ovarian transcriptome (total base mean: 31,729,678). Overall, mitochondrial genes were expressed significantly more highly than nuclear genes. Previous studies have identified mitochondrial differences in various human tissues, in which the mitochondrial transcriptome abundance is directly proportional to the energy requirements of the given tissue. The mitochondrial transcripts in the heart have been shown to compose ~30% of the total mRNA, whereas adrenal, ovary, thyroid, prostate, testes and lung tissues make up only ~5% of the mitochondrial transcripts [28]. The elevated amounts of mitochondrial transcripts in sheep ovaries revealed that the expression of mitochondrial transcripts varies among not only tissues but also species. Our results further implied that sheep ovaries are a high-energy demand tissue compared with those of mono-ovulatory species, such as humans.

We observed that one or more mRNA libraries prepared from the same ovary resulted in variations in the amount of RNA and the level of gene expression, whereas the libraries prepared from the same RNA sample (i.e., mRNA derived from the same tube of RNA sample) were identical. Therefore, we considered the replicates derived from the same mRNA sample as being true replicates and those from different mRNA extracts but from the same ovary as being biological replicates, which were used for conducting differential expression analyses. There were 12 different classification categories, including within-breed effect of diet, differences between breeds with diet as a secondary factor, comparisons to identify genes associated with the flushing diet and differences between breed groups without considering the effect of diet, as detailed below. On the basis of the differential expression analyses, we compiled a list of potential candidate genes (see Table 2 below).

**Table 2:** List of 20 candidate genes based on differential expression analysis. Differential expression level of the genes for different comparisons are available in the additional files listed under “Additional files (AF)” column

Genes	count	Chromosome	Gene Name	Description	Additional files (AF)
ENSOARG00000012224	612.7	X	BMX*	BMX non-receptor tyrosine kinase	AF3, AF4, AF5
ENSOARG00000008913	73.4	21	CSRP3	Cysteine and glycine-rich protein 3	AF3,AF4, AF5
ENSOARG00000000531	2034	5	ARGHEF18*	Rho guanine nucleotide exchange factor 18	AF3, AF4, AF5, AF6
ENSOARG00000012165	129.6	5	ADAMTSL5	ADAMTS-like protein 5	AF3, AF4, AF5
ENSOARG00000001346	143.7	21	CST6	Cystatin E/M	AF3, AF4, AF6
ENSOARG00000005014	521.7	5	ABLIM3	Actin binding LIM protein family, member 3	AF3, AF4, AF5
ENSOARG00000012206	1081.7	14	CA5A	Carbonic anhydrase VA, mitochondrial	AF3, AF4, AF5
ENSOARG00000001554	423.2	19	FBLN2	Fibulin 2	AF3, AF4, AF5
ENSOARG00000005722	10,459.6	3	EPAS1	Endothelial PAS domain protein 1	AF3, AF4, AF5
ENSOARG00000017011	1248.6	9	CYP7B1	Cytochrome P450, family 7, subfamily B, polypeptide 1	AF3, AF4, AF5
ENSOARG00000017264	381.2	20	BMP6	Bone morphogenetic protein 6	AF5
ENSOARG00000008743	889.1	19	CNTN4	Contactin 4	AF5
ENSOARG00000019163	85.8	15	HBB*	Hemoglobin subunit beta	AF6
ENSOARG00000002865	446.6	6	MEPE	Matrix extracellular phosphoglycoprotein	AF6
ENSOARG00000005888	166.1	2	PAPPA	Pregnancy-associated plasma protein A, pappalysin 1	AF3, AF4, AF5
ENSOARG00000018396	9244	17	SCARB1	Scavenger receptor class B, member 1	AF3, AF4, AF5
ENSOARG00000003867	12,811.7	18	CYP11A1	Cytochrome P450, family 11, subfamily A, polypeptide 1	AF4, AF5
ENSOARG00000014253	311.1	17	SLC7A11	Solute carrier family 7 (anionic amino acid transporter light chain, xc-system), member 11	AF5
ENSOARG00000000345	3872.5	15	GRAMD1B	GRAM domain containing 1B	AF3, AF4, AF5
ENSOARG00000011449	61.8	16	PRLR	Prolactin receptor	AF3, AF4, AF5
ENSOARG00000014199	10,852.6	2	PLIN2	Perilipin 2	AF3, AF4

When comparing the within-breed effect of diet, we did not identify any differentially expressed genes (DEGs) in Finnsheep that were associated with diet; however, 118 genes (71 upregulated in the flushing group, Additional file 3) from Texel were differentially expressed. Similarly, 25 genes (4 upregulated in the flushing group) were differentially expressed between the two F1 crosses fed a different diet. The majority of genes were upregulated in Texel because of the effect of the flushing diet. However, most of the DEGs were downregulated in the flushing group of F1 crosses. In general, the flushing diet had a profound effect in Texel, as shown by the comparatively higher number of DEGs in comparisons both within and between breeds. Additionally, Finnsheep were the least responsive to diet. The minor effect of the flushing diet on the litter size of Finnsheep ewes has also been previously reported in a feeding experiment^9^.

In comparisons between breed groups with the introduction of diet as a second factor, highest number of genes were differentially expressed between Texel and F1 crosses (Additional file 4). A total of 38 genes were differentially expressed between the pure breeds, with only four genes (*CST6*, *LPAR2*, *ENSOARG0000000531* (*ARGHEF18*) and *DIS3L2*) upregulated in Finnsheep. Sixty-eight DEGs were identified between Texel and the F1 crosses, and 44 were upregulated in Texel. Similarly, only five genes (*ENSOARG00000002559* (*PCDH11X*), *ENSOARG0000004833* (*RUFY1*), *CSRP3*, *DPP10* and *CA5A*) were differentially expressed between Finnsheep and F1 crosses, with *CA5A* and *CSRP3* upregulated in F1 crosses. In general, the DEGs were upregulated (34 in Finnsheep vs. Texel and 44 in Texel vs. F1 crosses) in Texel ewes.

In comparisons between breeds, using the flushing diet group subset, the largest numbers of DEGs were found between Finnsheep and Texel, followed by the Texel and F1 crosses, and the smallest number of DEGs was found between Finnsheep and F1 crosses (Additional file 5). Altogether, 600 genes were differentially expressed between Finnsheep and Texel, and 305 genes were differentially expressed between Texel and F1 crosses. In those two conditions, most genes (487 in Finnsheep vs. Texel and 249 in Texel vs. F1 crosses) were upregulated in Texel. Additionally, 47 genes were differentially expressed between Finnsheep and F1 crosses, 18 of which were upregulated in Finnsheep. Nine genes, *BSPH1*, *C17orf67*, *SERPINE1*, *CSRP3*, *CA5A*, *NELL1* (two paralogs), *ENSOARG00000004833* (homologous to *RUN* and *FYVE* domain-containing protein 1 – *RUFY1*) and *KRT8*, were found to be present in both the Finnsheep vs. F1 cross and Texel vs. F1 cross comparisons. Interestingly, all genes except *RUFY1* were upregulated in the F1 crosses and downregulated in both Finnsheep and Texel. These differences in gene expression revealed that, overall, the flushing diet had a greater effect on Texel than the other breed groups. The smallest number of DEGs between Finnsheep and the F1 crosses demonstrated that the F1 crosses responded more similarly to Finnsheep than Texel.

Comparisons involving control diet samples revealed only three DEGs (*CST6*, *MEPE* and *ENSOARG00000019163* (*HBB*)) between the pure breeds, with *CST6* and *MEPE* upregulated in Texel (Additional file 6). Similarly, only two genes were differentially expressed between Finnsheep and the F1 crosses, among which *HBB* was upregulated in Finnsheep, and *LMOD2* was downregulated in the F1 crosses. Unexpectedly, 57 genes were differentially expressed between Texel and the F1 crosses. In F1 crosses, in contrast to other groups, the ratio of upregulated genes was comparatively higher than that in Texel. These results showed that fewer genes were differentially expressed in the control subsets of ewes compared with those maintained on a flushing diet.

Three genes, *CST6*, *MEPE* and *HBB*, which were differentially expressed between the purebreds receiving the control diet, appeared to be candidate genes for prolificacy traits. Notably, the *CST6* gene was also differentially expressed in the diet comparisons. This gene is involved in processes such as anatomical structure morphogenesis, epidermis development and negative regulation of endopeptidase activity. Previous studies have indicated its role in the differentiation process of the epidermis [29, 30]. Owing to the inhibitory role of the gene, the lower level of *CST6* expression most probably promotes ovulation. Whereas *CST6* expression was downregulated in Finnsheep and the F1 crosses compared with Texel, the flushing diet appeared to minimize the expression of this gene in Texel, because it was downregulated in the Texel group receiving the flushing diet (within breed diet comparisons). To date, studies examining the functional aspects of *MEPE* have focused on humans and have highlighted a role in bone proliferation and differentiation [31–33]. Both *CST6* and *MEPE* were downregulated in Finnsheep and upregulated in Texel. One explanation for the differential expression of these genes might be that the higher level of cell differentiation promoted by these genes may be associated with greater follicle numbers. Similarly, the differential expression pattern may also be associated with the difference in the progression of the follicular phase of the oestrus cycle. Thus, the follicular growth phase might progress faster in Finnsheep than in Texel because cellular differentiation occurs only after proliferation.

### GO and KEGG pathway analysis of differentially expressed genes

**Fig. 2:**
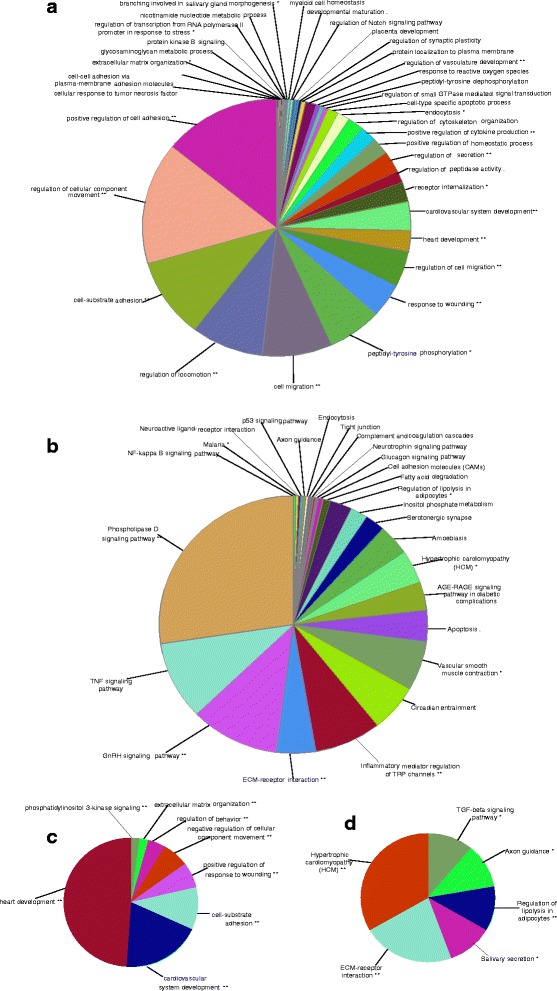
Gene Ontology and pathway annotations of differentially expressed genes. **a** GO terms associated with genes that were differentially expressed between the flushing subsets of Finnsheep and Texel. **b** KEGG pathways associated with genes that were differentially expressed between the flushing subsets of Finnsheep and Texel. **c** GO terms associated with genes that were differentially expressed between the flushing subsets of Texel and the F1 crosses. **d** KEGG pathways associated with genes that were differentially expressed between the flushing subsets of Texel and the F1 crosses

From the list of 600 genes differentially expressed genes between Finnsheep and Texel receiving the flushing diet, 435 genes were associated with 220 Gene Ontology (GO) terms, which were further clustered into 36 GO groups on the basis of a Kappa score threshold of 0.4 (Fig. 2A, Additional file 7). The largest number of genes (*n* = 49) was associated with cardiovascular system development, and the largest percentage of genes (~20%) was associated with branching involved in salivary gland morphogenesis. The majority of GO terms were associated with developmental processes, such as cardiovascular system development, cell migration, cell-substrate adhesion, heart development, regulation of cellular component movement and regulation of locomotion. In addition, several pathways, such as glycosaminoglycan metabolic process, nicotinamide nucleotide metabolic process, peptidyl-tyrosine dephosphorylation and peptidyl-tyrosine phosphorylation, were associated with the DEGs. Because most of the genes were upregulated in the Texel group, we concluded that the flushing diet promotes developmental processes in Texel. Similarly, Kyoto Encyclopedia of Genes and Genomes (KEGG) pathway analysis revealed 85 pathways that were clustered into 26 groups (Fig. 2B, Additional file 8). Enriched KEGG pathways included a number of signalling pathways (phospholipase D signalling, TNF signalling, GnRH signalling, NF-kappa B signalling, p53 signalling, neurotrophin signalling, and glucagon signalling pathways), extracellular matrix (ECM)-receptor interaction, circadian entrainment, vascular smooth muscle contraction, serotonergic synapse, regulation of lipolysis in adipocytes, fatty acid degradation and axon guidance.

Among 305 DEGs between the subset of Texel and F1 crosses receiving the flushing diet, GO and KEGG annotations were available for 226 genes. Those 226 genes were associated with 41 GO terms and 9 KEGG pathways, which were further clustered into 8 GO terms (Fig. 2C, Additional file 9) and 6 KEGG pathways (Fig. 2D, Additional file 10). The majority of the GO terms were associated with heart development (angiogenesis, heart morphogenesis, heart contraction and cardiovascular development). Similarly, the KEGG pathways included hypertrophic cardiomyopathy (HCM), ECM-receptor interaction, salivary secretion, regulation of lipolysis in adipocytes, axon guidance and the transforming growth factor (TGF)-beta signalling pathway.

### miRNA expression in ovaries

After trimming and size selection, 595,743,784 miRNA-seq reads were obtained for mapping, of which 291,954,185 were aligned to the sheep reference genome, with an average of 7.9 million reads per sample (Additional file 1: Table S5). In addition, each sample contained approximately 27,000 precursor miRNAs and 5.8 million mature miRNA sequences. The number of known sheep miRNAs ranged from 101 to 126, with an average of 112 miRNAs per sample and at least 5X coverage. The base mean value was at least 10 reads for a given miRNA, and altogether 342 miRNAs were expressed across all samples, 238 of which were novel miRNAs (Additional file 11). Oar-miR-10b was the most highly expressed miRNA, and the top ten miRNAs included two novel sheep miRNAs homologous to miR-486 (novel_26_614) and miR-92a (novel_10_75).

Among 12 comparisons that were similar to the mRNA data, significantly differentially expressed miRNAs were found in only two different diet conditions for the pure breeds. Three novel sheep miRNAs (Novel_1_45, Novel_21_499 and Novel_9_845) were differentially expressed between Finnsheep and Texel receiving a normal diet (Additional file 12). Nonetheless, a homology-based sequence search revealed that all three novel sheep miRNAs are conserved in other mammals, such as humans and cattle. The three novel sheep miRNAs are homologous (with 100% sequence coverage and identity) to miR-6529, miR-192 and miR-151, respectively. miR-6529 was upregulated in Texel, whereas miR-192 and miR-151 were upregulated in Finnsheep. Among eight miRNAs that were differentially expressed between pure breeds maintained on a flushing diet, four were upregulated in Finnsheep (Additional file 13). Moreover, six of those miRNAs were novel, and a BLAST search revealed the homology of novel sheep miRNAs to miR-2285 m-3 (novel_10_67), miR-326 (novel_15_269), miR-130a (novel_15_279), miR-2284 (novel_7_797), miR-1468 (novel_X_868) and miR-361 (novel_X_881) in mammals.

### Identification and annotation of novel miRNAs

Filtering out of miRNAs with fewer than 10 reads resulted in the retention of 238 of 933 novel miRNAs with distinct chromosome locations. Similarity searches of those 238 miRNAs against the cattle database resulted in 159 known miRNAs in cattle (Additional file 14). The remaining 79 novel miRNAs were queried against the human database, and none were detected. Thus, 79 novel miRNAs without any homologues were classified as truly novel miRNAs. Unlike known miRNAs, which were primarily expressed on chromosome 18 (*n* = 51), the largest number of novel miRNAs (*n* = 35) was expressed on chromosome X. Of the novel miRNAs, 13 were associated with chromosome 18.

### Effects of genetic variants

**Fig. 3:**
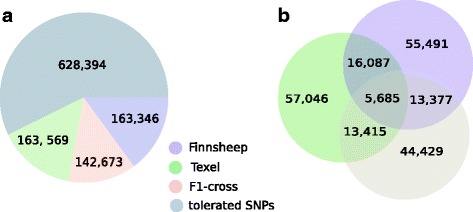
Distribution of deleterious SNPs in the three groups of sheep populations. **a** We identified 469,588 deleterious SNPs, which were classified into three breed groups. **b** Among the deleterious SNPS, 5685 SNPS were common to all three groups

Variant effect predictor (VEP) processed 175,598,436 variants from the aligned mRNA-seq data, of which approximately 69% were novel variants. Additionally, 94.3% of all processed variants were predicted to alter only the sequence, thus resulting in 13,475,005 variants that were classified as single-nucleotide variant (SNV; 12,922,871), substitution (432,083), deletion (65,620) and insertion (54,431) types. Further classification of the variants on the basis of the variant effects resulted in 23 categories with intron variants associated with 36.1% of the variants, followed by intergenic variants (20.7%), downstream gene variants (18.6%), upstream gene variants (8%) and missense variants (6.7%) as the top five consequences. We also identified variants associated with coding sequences, including 1,955,207 variants that were further classified into seven different categories, among which missense variants were the most common (Additional file 15). Sorting Intolerant From Tolerant (SIFT) analysis predicted 1,097,982 SNPs with non-synonymous effects, among which (469,588) 42.8% were deleterious, and the rest were tolerated (Fig. 3A). The number of SNPs was more closely related to the size of the chromosome than to the number of genes per chromosome. In general, the numbers of SNPs was higher in the N- and C- termini of functional peptides, with 211,160 SNPs in the N-terminus and 202,853 in the C-terminus. The distributions of deleterious SNPs were similar in Finnsheep and Texel, which shared 163,346 and 163,569 SNPs, respectively, whereas the F1 crosses had 142,673 SNPs (Fig. 3B). We combined the SNPs at the breed level (by extracting SNPs from all individuals belonging to a particular breed group) and found that Finnsheep had 90,640 SNPs, Texel had 92,233 SNPs, and the F1 crosses had 76,906 SNPs. Among these SNPs, 27,383 were common to all three breeds (Fig. 3B). Similarly, Finnsheep, Texel and the F1 crosses had 28,829, 30,330 and 18,957 private SNPs, respectively.

We observed in detail the variants of four major candidate genes (*GDF9*, *BMP15*, *BMPR1B* and *B4GALNT2*). Altogether, *GDF9* had 506 SNP positions, of which 82 were non-synonymous mutations. Among the 82 non-synonymous SNPs, 52 were predicted by VEP as being deleterious (substitutions that change gene function) and were located at 15 unique positions in the gene between 5:41,841,072 and 5:41,841,965 on chromosome 5. None of the previously studied mutations, such as FecGH(S395F), FecTT(S427R) and FecGE(F345C), were present in any of the samples. Interestingly, the mutation V371 M (rs403536877; C-T transition at 5:41,841,285) was present in five Finnsheep and four F1 crosses but was not present in Texel. Unexpectedly, the SNP in one of the ewes (including two additional replicate samples) from the F1 crosses was homozygous, thus indicating that the ancestry of the animal was more than 50% Finnsheep (not supported by pedigree or SNP data) or that the mutation also occurred with a low frequency in the Finnish population of the Texel breed. The V371 M substitution, which is also present in Cambridge, Belclare and Norwegian White sheep, has been suggested to improve fertility and to have originated from Finnsheep [34–37].

In *BMP15*, 17 out of 138 SNPs were non-synonymous, and none of them were significant. Although *BMPR1B* had the largest number of SNPs (*n* = 2245) among the three candidate genes, only 33 were non-synonymous, and none of these SNPS were deleterious. For *B4GALNT2*, only 168 SNPs were predicted, but none of them were non-synonymous. None of these genes were differentially expressed in our comparisons (see also [19, 38]). Additionally, the gene expression levels of these candidate genes were comparatively lower in our samples. One explanation for this finding may be that these genes are stage-specific and may be highly expressed at some point during a later stage of the oestrus cycle.

In addition to four major genes known to be associated with the ovulation rate, we assessed SNPs in three genes that were differentially expressed between the pure breeds. We observed a SNP (rs413226920; D129E) in the *HBB* gene, which was expressed in three Finnsheep and two F1 crosses. The SNP was homozygous in Finnsheep and heterozygous in the F1 crosses. We observed additional deleterious SNPs in *HBB* and *MEPE*, but the number of samples with SNPs was too low (> 3) to determine their significance. Only some of the Finnsheep ewes were identified as carrying mutated (*HBB*, *n* = 3) and *GDF9* (*n* = 5) genes, which are associated with an increased ovulation rate, thus further supporting the presence of two different lines (high and low) in Finnsheep [38]. The presence of these mutations in some F1 crosses and in breeds derived from Finnsheep demonstrates that prolificacy traits in Finnsheep are highly heritable.

Genome Analysis Toolkit (GATK) analysis revealed 22,607 SNPs in the miRNA-seq data, of which only 9177 were classified as SNVs by VEP annotation (a summary of the miRNA variant analysis can be found in Additional file 16). We also found that 373 missense mutations were present in the miRNA sequences, of which 193 were predicted to be deleterious, and the remainder were tolerated on the basis of SIFT prediction. We did not find any correlation between chromosome size and the number of miRNA variants. The largest number of variants (8880) was present on chromosome 18. A close-up view of chromosome 18 revealed a peak of non-coding genes towards the 3′-end, where the majority of the miRNAs were located (Additional file 1: Fig. S3). All 51 miRNAs located within a 194-kb region (64,466–64,600 kb) of chromosome 18 were expressed in our samples. A large cluster of miRNAs was identified on chromosome 18 of the sheep genome, which also contained the largest number of miRNAs. Interestingly, all known miRNAs from chromosome 18 were expressed in the data, with two copies of miR-496. The orthologues of this cluster are present in other mammals, such as humans (Chr14), gorillas (Chr14), dogs (Chr8), horses (Chr24), rats (Chr6) and mice (Chr12). Not only the miRNAs but also the nearby genes were similar among mammals. The homologous mouse miRNAs were expressed exclusively from the maternal chromosome [39]. More specifically, they were located within the parentally imprinted regions *Dlk1-Gtl2*. This region, which is differentially methylated during embryonic development, lies within a parentally imprinted chromosomal area, *Dlk-Dio3*, in humans and is known to be highly important in development [40]. From this cluster, mir-376a and mir-376c are known to potentially target the 3′ untranslated region (3’UTR) of *IGF1R*.

### Integrated analysis of differentially expressed mRNAs and miRNAs

**Fig. 4:**
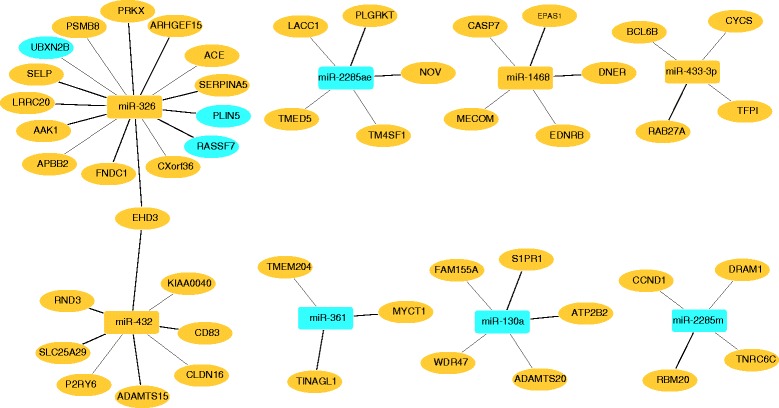
Integrated mRNAs and miRNAs that were differentially expressed between Finnsheep and Texel maintained on a flushing diet. Upregulated mRNAs and miRNAs are shaded in light blue, and downregulated mRNAs and miRNAs are shaded in orange

Three genes and miRNAs each were differentially expressed between Finnsheep and Texel receiving the control diet, but none of the three genes were predicted to be targets of the three miRNAs. However, 48 genes were in the list of targets predicted for 8 miRNAs, which were differentially expressed between pure breeds maintained on the flushing diet (Fig. 4). Among the 8 miRNAs, miR-326 had the most targets (*n* = 15), whereas miR-361 had only three targets. From the list of 48 target genes of the eight miRNAs, only three genes, *UBXN2B*, *PLIN5* and *RASSF7*, were upregulated in Finnsheep; the remainder (*n* = 45) were upregulated in Texel. Moreover, all three upregulated genes were targets of miR-326. In particular, *PLIN5* plays an important role in lipid metabolism and is highly expressed in tissues such as the heart and liver, which have high rates of fatty acid oxidation [41, 42]. miRNAs destabilize and degrade their target mRNA, thus preventing translation [43–45]. According to this principle, we anticipate an inverse relationship between the miRNAs and their target genes. Four miRNAs, miR-2285ae, miR-361, miR-130a and miR-2285 m, displayed a perfectly inverse relationship with their target genes, i.e., all four miRNAs were downregulated within the Texel flushing group, and their target genes were upregulated. However, three of the interactions that involved miR-1468, miR-433-3p, and miR-432 exhibited a clear direct relationship, and 12 of the 15 target genes of miR-326 had a direct relationship. These results suggest that in addition to the well-known mRNA-miRNA inverse relationship, there may be other ways by which miRNAs regulate their target genes.

### Results from validation experiments

**Fig. 5:**
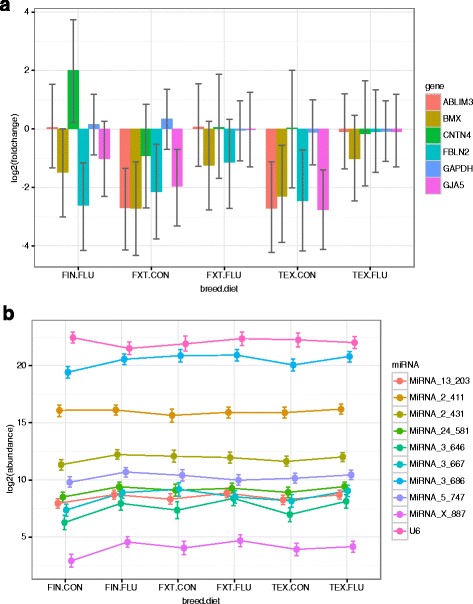
qPCR validation of mRNAs and miRNAs. **a** The threshold cycle (ct) values of mRNA expression were used to compute the fold changes to measure differential expression, **b** whereas for miRNAs, only the expression status was assessed

The expression levels of five randomly selected genes that were differentially expressed between Finnsheep and Texel ewes maintained on a flushing diet and 10 miRNAs from the studied samples were analysed by qPCR. The qPCR results for gene expression were largely consistent with the RNA-seq results, whereas the expression profiles of all tested miRNAs strongly suggested that all novel miRNAs from our list were of ovine origin (Fig. 5).

*GDF9* screening using Sanger Capillary PCR sequencing in 31 Finnsheep and 32 Texel ewes detected three polymorphic sites, including the known variation at rs403536877 c.1111G > A, which leads to V371 M at protein sequence position 5:41,841,285. This prolific mutation did not segregate in the present sample set of Texel, but in Finnsheep, the frequency of the *GDF9* c.1111G > A –mutation was 0.70, thus indicating that the identified SNP variation in Finnsheep and F1 crosses in the RNA-seq data was valid.

## Conclusions

Here, we provide the first report of sheep F1 crosses in RNA-seq-based studies, including a larger number of biological replicates, greater sequencing depth and more phenotypic evaluations than reported in earlier studies. Our analysis of the RNA-seq data from two globally important breeds with contrasting fertility traits revealed important features regarding the ovarian transcriptome landscape. Our results indicated that important markers (such as SNPs that contribute to the V371 M mutation in *GDF9*) can be easily inherited over generations, thus increasing the commercial value of these genetic markers and the Finnsheep breed itself. Our findings provide strong insights into the importance of the flushing diet to increase sheep reproduction, particularly in breeds with low reproductive traits. We found that Texel, a breed with a comparatively smaller litter size, was genetically responsive to the flushing diet, whereas Finnsheep were non-responsive on the basis of the absence of DEGs between flushing versus the control diet (within-breed). Unexpectedly, whereas the genotypes place the F1 crosses as a true hybrid of Finnsheep and Texel, gene expression patterns revealed that the F1 crosses were more similar to Finnsheep. The three differentially expressed genes between Finnsheep and Texel receiving the control diet may be candidate genes for prolificacy. Moreover, the most highly expressed genes were dominated by mitochondrial genes, thus illustrating that the ovaries are organs with a high energy demand in multiparous species. We identified a cluster of 51 miRNAs on chromosome 18, and a follow-up analysis of other reproductive tissues was required to characterize whether such clusters were tissue-specific. The identification and characterization of miRNAs that are differentially expressed between pure breeds should facilitate understanding of the regulatory roles of miRNAs during folliculogenesis in these breeds. Additionally, the novel miRNAs should enrich the relatively scarce resources available for sheep miRNAs. Future research investigating the roles of other important reproductive tissues, such as endometrium and *corpus luteum* from the same individuals but at different stages of the oestrous cycle, will allow elucidation of the roles of tissue-specific gene expression.

## Methods

### Animals and the feeding experiment

The feeding experiment was performed at Pusa farm in Urjala, Finland. All procedures for the feeding experiment and sheep sampling were approved by the Southern Finland Animal Experiment Committee (approval no. ESAVI/5027/04.10.03/2012). Non-pregnant and sexually mature ewes that were tested to be free of Maedi–Visna were obtained from commercial sheep farms. A total of 31 ewes were included in the experiment: 11 Finnsheep ewes, 11 Texel ewes and 9 Finnsheep-Texel F1 crosses. Because the response to the flushing diet is influenced by the age of the ewe, breed, body condition and stage of the breeding season [26, 46–53], we designed our experiment by keeping half of the ewes on a flushing diet to study how nutrition contributes to different breeds with varying prolificacy. In the feeding experiment, we applied typical Finnish sheep farm management practices. The details of the feeding experiment are provided as supplementary methods (see Additional file 1).

### Tissue, blood sampling and DNA/RNA extraction

The oestrous cycles of the ewes were followed by progesterone hormone profiling. The blood samples for the progesterone measurements were started 12 days after the arrival of rams at the sheep shed. All blood samples (5 ml, heparinized vacuum tubes) were collected in the morning from the jugular vein at 1- to 7-day intervals from each ewe, depending on the progesterone concentrations of the previous measurements. The blood was cooled, transferred to the laboratory within 3 h and analysed the same day. If the progesterone level was decreasing rapidly, the ewe underwent surgery the next day to capture the follicular growth phase. In addition, the oestrous cycles were monitored with retrospective FSH and anti-Müllerian hormone (AMH) measurements. To study the effects of the diets, the levels of insulin-like growth factor 1 (*IGF-1*), glucose, urea, albumin, and insulin were also measured during the experiment.

Because a follow-up of the ovaries was not possible with ultrasound, the oestrous cycles were monitored by daily progesterone measurements (Additional file 2). A sharp decrease in progesterone resulted in removal of the ovary the following day to obtain the follicular growth phase. During surgery, both ovaries were palpated, and the larger (and more active) one was removed. For surgery, the ewes were sedated, and local anaesthesia, postoperative analgesia and antibiotics were used. The numbers of follicles and corpora lutea (CLs) were counted in the removed ovary, and the ovary was photographed. Immediately after removal, the ovary was washed with physiological saline, and the middle part of the ovary with blood vessels and all visible CLs were removed. Thus, the biopsies included the remaining ovary tissue, which was not homogenous. CLs were collected separately from the remaining ovaries of the same ewes after establishment of pregnancy (data not shown). Ovaries were cut into a number of sections (20–30 mg) in RNAlater and stored in RNAlater reagent (Ambion/Qiagen, Valencia, CA, USA), per the manufacturer’s instructions, before tissues were transported to the laboratory. Tissue samples were stored at −80 °C until extraction. Total RNA, including small RNAs, was extracted using an RNeasy plus mini kit (Qiagen, Valencia, CA, USA) according to the manufacturer’s protocol (see Additional file 1 for the RNA extraction details). The RNA concentration and RNA integrity number (RIN) were measured using a Bioanalyzer 2100 (Agilent Technologies, Waldbronn, Germany). Blood samples were collected in EDTA tubes, and genomic DNA was extracted for SNP genotyping using the standard phenol-chloroform method [54].

### Ovine HD BeadChip SNP genotyping

The animals were divided into experimental groups on the basis of their pedigree records (purebred Finnsheep and Texel and their F1 crossbreds). To obtain an overview of the genetic relatedness of the animals and to assure the breed status, the samples were genotyped using Illumina Ovine 700 K SNP BeadChips (Illumina, USA). Genotyping was conducted at the Finnish Institute of Molecular Medicine (FIMM), Helsinki, Finland. All genotypes were called using GenomeStudio software v2011.1, and SNPs that failed to meet any of the following criteria were discarded: (1) low success rate (*n* = 3760); (2) low intensity (*n* = 2126); (4) uncertain clustering (*n* = 4635) and (5) too many clusters (*n* = 3571). Altogether, 606,006 SNPs that passed the initial quality control (QC) assessments were used to calculate the pairwise identity by distance (IBS) of 31 samples that were used for RNA-seq with Plink v1.90b3v [55].

### RNA library preparation and sequencing

Library preparations and sequencing were performed at FIMM. RNA libraries were prepared using TruSeq RNA Acess library kit (Illumina, Inc., San Diego, CA, USA) according to the Illumina TruSeq® Stranded mRNA Sample Preparation Guide (part #15031047). Unique Illumina TruSeq indexing adapters were ligated to each sample during the adapter ligation step, and subsequently several samples were pooled in one flow cell lane. The high quality of the libraries was confirmed with an Agilent Bioanalyzer 2100 (Agilent Technologies, Waldbronn, Germany), and the concentrations of the libraries were quantified via Quibit® fluorometric quantitation (Life Technologies, Santa Clara, CA, USA). Only high-quality libraries were sequenced. The samples were normalized and pooled for automated cluster preparation, which was conducted with an Illumina cBot station. The samples were sequenced with an Illumina HiSeq 2000 instrument with TruSeq v4 sequencing chemistry. Paired-end sequencing with a 2 × 100-bp read length was used for mRNA and single-end sequencing with 1 × 50-bp read length for the miRNAs. Additional mRNA and miRNA libraries for one of the samples from the Finnsheep flushing, Texel flushing and F1 flushing groups were sequenced. Additionally, one of the mRNA libraries from the F1 control group was sequenced three times. Hence, altogether, we obtained sequence data for 39 mRNA-seq and 37 miRNA-seq samples.

### Processing of the sequencing data

The overall quality of mRNA- and miRNA-seq reads was inspected using the FastQC tool (http://www.bioinformatics.babraham.ac.uk/projects/fastqc/). Cutadapt tool [56] was used to trim all adapters and to perform size selection of the sequence data. In addition, low-quality reads and reads shorter than 20 bp were discarded from the raw mRNA-seq reads. Similarly, from the miRNA-seq data, reads with lengths ranging from 16 to 26 nt were selected for downstream analyses. Trimmed reads were reassessed to ensure that the data were free from noise.

Clean raw reads from both mRNA-seq and miRNA-seq data were mapped against the ovine reference genome (Oarv3.1) and Ensembl (Ensembl release 76) annotations [57, 58]. mRNA-seq reads were mapped using the Tophat2 v. 2.0.14 program [59, 60], whereas the Bowtie v. 1.1.1 program [61] was used to map miRNA sequences against the ovine reference genome. MiRDeep2 [62] was used to identify known miRNAs and to predict novel miRNAs. The overall RNA quantification was conducted using the CAP-miRSeq [63] tool to better understand the quality of the miRNA-seq experiment. The overview of the bioinformatics workflow is presented graphically in additional information 1 (Additional file 1: Fig. S4).

### Differential expression analysis

To examine the possible influence of the flushing diet, we implemented differential gene expression analysis within and between breeds by incorporating diet as a second factor. In addition, to obtain an overview of genetic differences between pure breeds, we performed differential gene expression analyses on the subset of the controlled diet group. DESeq2 [64] was used to test DEGs and miRNAs within and between breeds for 12 different comparisons. The ovine transcriptome was downloaded from the Ensembl database, and all of the bam files that originally mapped to the ovine reference genome were queried against the transcriptome to count the reads belonging to exonic regions. The Python-based software htseq-count [65] was used to produce counts from the aligned reads. Adjusted *p*-values of 0.05 and 0.10 were used to generate the list of significant DEGs and miRNAs, respectively. Because predicted novel miRNAs have only genomic coordinates and can differ among samples, comparing a large number of samples would be difficult. However, a true novel miRNA is often detected in multiple samples. We implemented a strategy to merge a commonly detected novel miRNA across samples if their start/end coordinates overlapped by at least 80%. A new genomic coordinate would be created for these miRNAs by using the outermost coordinate. We observed that the most commonly detected miRNAs had the same or very similar coordinates, thus providing further verification of a true novel miRNA. We used this approach as implemented in the CAP-miRSEQ pipeline, and all novel miRNAs and their corresponding genome coordinates were listed in a table. All DEGs were used for GO annotations and KEGG pathway analyses.

### Prediction of miRNA target genes

TargetscanHuman release 7.1 [66] was used to retrieve all gene targets of differentially expressed miRNAs. The resulting target genes belonged to the human genome, and we were interested only in DEGs. Thus, human orthologues of all DEGs for a given comparison were retrieved using the bioconductor package in BioMart [67, 68]. Finally, the differentially expressed human orthologues were searched in the list of predicted target genes that had a cumulative weighted context^++^ score greater than −0.29.

### Gene annotation

Ensembl contained 175,812 GO terms for 18,590 genes, among which catenin (cadherin-associated protein; *ENSOARG00000002885*) was associated with the largest (205) number of GO terms. Protein binding was the most common GO term amongst all known sheep genes and DEGs. Both the GO annotations and KEGG pathways were analysed in Cytoscape [69] using the ClueGO [70] plugin. We set the initial criteria of at least five genes and 5% of genes to be present in the list of DEGs for identifying GO terms. Similarly, KEGG pathways representing at least four DEGs and 4% of the given pathway were retrieved. GO terms covering five different levels (levels 3–8) were grouped on the basis of similar associated genes with a Kappa score threshold of 0.4. GO terms and pathways for genes that were differentially expressed in two (Finnsheep vs. Texel with the flushing diet and Texel vs. F1 crosses with the flushing diet) of the 12 comparisons were identified. We noticed that many sheep genes were missing annotations from the ClueGO source files available for sheep. Therefore, all of the DEGs were grouped, and the unique Ensembl IDs were selected to determine the number of genes that lacked annotations. All genes without annotations available in the ClueGO source files were queried using the Ensembl BioMart server [71] to retrieve three additional features: Entrez ID, transcript ID and protein ID. The ClueGO source files were manually updated by adding those features. With that approach, comparatively high numbers of genes were annotated. ClueGO allows users to define certain thresholds and configurations to identify significant ontologies and pathways. Similar GO terms and pathways were grouped together using a Kappa score threshold of 0.4. A right-side hypergeometric test with a Bonferroni step-down correction method was used to identify significant terms and pathways. A minimum of three and a maximum of eight GO levels were included in our analysis. To be considered as an enriched term or pathway, at least one gene and at least 5% of genes should be expressed.

Novel miRNAs with baseMean (mean expression between all samples, as explained in the DESeq2 bioconductor package) ≥ 10 were annotated using a BLAST search against the human and cattle sequence database. All novel sheep miRNAs with homology to other mammalian species were annotated on the basis of homologous miRNAs, and novel miRNAs without reasonable similarity to other species were validated by qPCR.

### Gene variant analysis

SAMtools [72] was used to call SNPs from sorted bam files of the mRNA-seq data. After SNP calling, low-quality SNPs (quality less than 10) and SNPs that appeared at more than two times the average depth coverage of the samples were removed. The average depth coverage for each sample was calculated using bedtools [73], and the mean of the average depth coverage for all samples was used for filtration. In addition, SNPs within 3 bp and indels and clusters of indels separated by ≤5 bp were removed. The resulting SNPs were annotated using the standalone Ensembl VEP tool [74]. VEP predicts the consequence of variants on genomic regions and the locations of the variants. The VCF file consisting of the SNPs for all samples was used for the annotation against the ovine genome. Sorting Intolerant From Tolerant (SIFT) [75] was incorporated into the analysis to predict whether the amino acid substitution would affect the protein function [75]. SNP mutations in four major candidate genes, i.e., *GDF9*, *BMP15*, *BMPR1B* and *B4GALNT2,* which have been studied previously in other sheep breeds, were assessed.

miRNA variants were analysed using GATK [76], which forms part of the CAP-miRSeq pipeline. The SNVs located in the seed regions of the mature miRNA were considered for further annotation. The miRNA variants were again annotated using VEP, and the analysis steps were similar to those used for the mRNA-seq data described above.

### Validation experiments

The expression levels of randomly selected genes and miRNAs were also assessed by qPCR. Total RNA was isolated from 39 ovarian tissue samples representing the two sheep breeds (Texel and Finnsheep) and treatment subgroups (flushing and control) using the AllPrep® DNA/RNA/miRNA kit (Qiagen, Hilden, Germany) according to the manufacturer’s instructions. The details of the RNA extraction are provided as supplementary methods (see Additional file 1).

Real-time PCR primers were designed on the basis of the mRNA sequences of the selected 5 candidate genes available in the GenBank database using Primer3 Express version 4.0.0 software [76] (http://primer3.wi.mit.edu//). Quantitative analysis of cDNA samples was performed using an ABI PRISM® 7000 sequence detection system (Applied Biosystems, Foster City, CA, USA). The PCRs were performed in a 20-μl reaction volume containing 13 μl of SYBR Green PCR master mix (Life Technologies, Helsinki, Finland). During each PCR, samples from the same cDNA source were run in duplicate. A universal thermal cycling parameter (10 min at 95 °C followed by 40 cycles of 15 s at 95 °C and 10 s at 60 °C) was used to quantify each gene of interest. The final quantitative analysis was performed using the ΔΔ C(t) method, and results are reported as the relative expression or n-fold difference in the calibrator (control group) after normalization of the transcript amount relative to the value of the endogenous control gene (GAPDH).

Total RNA enriched with miRNAs was isolated from the ovaries of all experimental animals by using an AllPrep® DNA/RNA/miRNA kit (Qiagen, Hilden, Germany) according to the manufacturer’s instructions. The cDNA synthesis was performed using a miScript® II RT kit (Qiagen, Hilden, Germany). The expression profiles of nine selected miRNAs (3–686, 2–411, 2–431, 5–747, 3–667, 24–581, 13–203, 3–646 and X-887) in addition to U6 as an endogenous control was performed with a miScript Sybr® green PCR kit used with primer assays (Qiagen, Hilden, Germany). The primers of all selected miRNAs were purchased from the same company (Qiagen, Helsinki, Finland). The PCR master mix was prepared using 12.5 μl of 2× QuantiTec Sybr green PCR master mix, 2.5 μl of 10× miScript universal primer, 2.5 μl of 10× primer assay set, and 5 μl of ddH2O added to 2.5 μl of cDNA template. The reaction was performed using a universal thermal cycling parameter program with initial heating at 95 °C for 15 min, followed by 40 cycles of 94 °C for 15 s, 55 °C for 30 s and a final extension at 70 °C for 30 s. Raw qPCR data for both the mRNA and miRNA samples were analysed using generalized linear mixed models based on lognormal Poisson error distribution as described in the R package MCMC.qpcr [77, 78].

A breed-wise validation was performed for the *GDF9* gene to confirm the population-level variation, particularly at position 5:41,841,285. This procedure was performed using genomic DNA with independent and randomly selected samples from the Texel and Finnsheep populations (31 and 32 individuals, respectively) by Sanger capillary direct PCR sequencing. The sequenced fragment was multiplied and sequenced by using the following PCR primers: 5’ GCC AGG ACA CTC ATG GTT TT’3 and 5’CTT CCA CCC TAA AAG GAA CC’3. The sequenced product size was 556 bp in length.

## Additional files

Additional file 1: This file includes supplementary methods, four supplementary figures (**Figure S1 – Figure S4**) and five supplementary tables (**Table S1 – Table S5**). **Table S1.** Ewes in the trial. **Table S2.** Compositions of the feed in the feeding trial. Units g/kg DM unless otherwise stated. **Table S3.** Effect of flushing on the body condition scores of the ewes. **Table S4.** Summary of the mRNA-seq data. Sample names in the first column also include breed and diet information. For example, TC_11E refers to sample 11E from the Texel breed receiving a control diet, and F1C_19A refers to sample 19A from the F1-cross of Finnsheep and Texel on a control diet. The number of genes (in the last column) includes all transcripts with at least 10 reads. One important observation is that the concentration of RNA in the samples does not necessarily affect the number of expressed genes. **Table S5.** Summary of the miRNA-Seq data. Sample names in the first column also contain the breed and diet information, similarly to **Table S4** in additional file 1. For each sample, we removed Illumina adapters and low-quality reads and used clean reads for downstream analysis. **Figure S1.** Relatedness analysis. (A) MDS plot based on (IBS) distances calculated using SNP genotype data. The same 31 ewes that were also employed for mRNA and miRNA sequencing were genotyped. (B) Principal component analysis (PCA) plot of the top 500 differentially expressed genes. **Figure S2.** Venn diagram showing the overlap of the top 500 genes among the breeds. **Figure S3.** Sheep chromosome 8 showing the peak representing the miRNA clusters identified in this study. **Figure S4.** Flowchart showing the integrated analysis of mRNA and miRNA data. (DOCX 251 kb)

Additional file 2: Summary of sheep phenotype records that also includes all of the different blood plasma measurements. (XLSX 25 kb)

Additional file 3: Within-breed diet effect. List of genes that were differentially expressed between the two diet conditions in Finnsheep, Texel and F1 crosses. (XLSX 30 kb)

Additional file 4: Between-breed diet effect. List of genes that were differentially expressed between Finnsheep and Texel; Finnsheep and F1 crosses; and Texel and F1 crosses with diet as a second factor. (XLSX 26 kb)

Additional file 5: Differential expression between flushing-diet sub-groups. List of genes that were differentially expressed between a subset of Finnsheep and Texel; Finnsheep and F1 crosses; and Texel and F1 crosses maintained on a flushing diet. (XLSX 144 kb)

Additional file 6: Differential expression between control-diet sub-groups. List of genes that were differentially expressed between a subset of Finnsheep and Texel; Texel and F1 crosses; and Finnsheep and F1 crosses maintained on a control diet. (XLSX 19 kb)

Additional file 7: GO annotation for genes that were differentially expressed between Finnsheep and Texel on a flushing diet. (XLSX 12 kb)

Additional file 8: KEGG pathways associated with genes that were differentially expressed between Finnsheep and Texel on a flushing diet. (XLSX 11 kb)

Additional file 9: GO annotation of genes that were differentially expressed between Texel and F1 ewes on a flushing diet. (XLSX 9 kb)

Additional file 10: KEGG pathways associated with genes that were differentially expressed between Texel and F1 ewes on a flushing diet. (XLSX 9 kb)

Additional file 11: List of all miRNAs (known and novel) expressed in our data with a base mean value ≥10 reads. (XLSX 202 kb)

Additional file 12: List of miRNAs that were differentially expressed between Finnsheep and Texel on a control diet. (XLSX 9 kb)

Additional file 13: List of miRNAs that were differentially expressed between Finnsheep and Texel on a flushing diet. (XLSX 9 kb)

Additional file 14: Annotation of novel miRNAs on the basis of cow and human homology. (XLSX 22 kb)

Additional file 15: Summary of SNP annotations from mRNA data using the Ensembl VEP tool. (XLSX 20 kb)

Additional file 16: Summary of SNP annotations from miRNA data using the Ensembl VEP tool. (XLSX 12 kb)
