# Supplementary material for: Integrated ovarian mRNA and miRNA transcriptome profiling characterizes the genetic basis of prolificacy traits in sheep (Ovis aries)
Source: BMC Genomics. 2018 Jan 29;19:104. doi: 10.1186/s12864-017-4400-4 (PMC5789708; doi:10.1186/s12864-017-4400-4)
Supplement: Supplementary file 1 — This file includes supplementary methods, four supplementary figures (Figure S1 – Figure S4) and five supplementary tables (Table S1 – Table S5). Table S1. Ewes in the trial. Table S2. Compositions of the feed in the feeding trial. Units g/kg DM unless otherwise stated. Table S3. Effect of flushing on the body condition scores of the ewes. Table S4. Summary of the mRNA-seq data. Sample names in the first column also include breed and diet information. For example, TC_11E refers to sample 11E from the Texel breed receiving a control diet, and F1C_19A refers to sample 19A from the F1-cross of Finnsheep and Texel on a control diet. The number of genes (in the last column) includes all transcripts with at least 10 reads. One important observation is that the concentration of RNA in the samples does not necessarily affect the number of expressed genes. Table S5. Summary of the miRNA-Seq data. Sample names in the first column also contain the breed and diet information, similarly to Table S4 in additional file 1. For each sample, we removed Illumina adapters and low-quality reads and used clean reads for downstream analysis. Figure S1. Relatedness analysis. (A) MDS plot based on (IBS) distances calculated using SNP genotype data. The same 31 ewes that were also employed for mRNA and miRNA sequencing were genotyped. (B) Principal component analysis (PCA) plot of the top 500 differentially expressed genes. Figure S2. Venn diagram showing the overlap of the top 500 genes among the breeds. Figure S3. Sheep chromosome 8 showing the peak representing the miRNA clusters identified in this study. Figure S4. Flowchart showing the integrated analysis of mRNA and miRNA data. (DOCX 251 kb) [file 12864_2017_4400_MOESM1_ESM.docx]

**Supplementary methods, supplementary references, supplementary figures S1-S4, supplementary tables S1-S5**

**Supplementary Methods**

**Feeding experiment**

The ewes were housed individually in 1.5 × 1.5-m pens so that they were able to see and smell one another. The bedding material was peat and straw, but because of hay spilling, only small amounts of straw or peat were needed during the experiment. The ewes arrived at the experimental site on 3 September 2012. Prior to the arrival, the ewes had been raised in a pasture away from the sight or smell of rams for at least 2 months. Two Finnsheep rams arrived at the sheep shed on 8 October 2012. The rams were used for synchronization and detection of oestruses. After their arrival, the ewes were able to see and smell the rams at all times. In addition, the rams were walked around the sheep shed three times per day between 8 and 21 October to ensure an effect.

During the three-week adaptation period, the ewes were fed timothy hay (9.3 MJ ME and 60 g crude protein/kg DM) and water *ad libitum*. The ewes were also given a mineral supplement (Lammas Hertta Muro, Manufacturer Suomen Rehu, Hankkija Oy, Hyvinkää, Finland) at a rate of 10 g/ewe per day. The mineral supplement contained the following: Ca 130 g, P 86 g, Na 75 g, Mg 60 g, Se 20 mg, Cu 10 mg, Fe 1600 mg, Zn 5150 g, Mn 4100 mg, I 150 mg, vitamin A 400,000 IU, vitamin D 120,000 IU and vitamin E 1,800 IU per kg. The feeding experiment was started on 24 September 2012. Each breed group of ewes was split between the two diets: normal (i.e., non-flushing) and flushing. As during the adaptation period, the non-flushing diet consisted of timothy hay provided *ad libitum* and the mineral supplement. The flushing diet included an additional 300 g of oats and 100 g of rapeseed meal (Krono 35, Manufacturer Suomen Rehu, Hankkija Oy, Hyvinkää, Finland) per day.

After their arrival at the experimental farm, the body condition score (BCS) and weight of the ewes were determined [1]. Subsequently, their BCS was followed at two-week intervals throughout the rest of the experiment. Ewes with a BCS of 3.75 or above did not receive oats. The concentrates were fed on the basis of their volume, and the daily portions of oats and rapeseed meal were split and fed at two time points during the day (morning and evening).

Representative feeds were sampled, and a pooled sample was sent for analysis. Hay fed during the adaptation period was analysed by using a near infrared reflectance spectroscopy (NIRS) method in the laboratory of Valio Ltd, and feed values were calculated according to Finnish Feed Tables (https://goo.gl/is2C2V). Other chemical analyses of the feeds were performed at the laboratory of Natural Resources Institute in Jokioinen, Finland. The laboratory has a quality system that follows the SFS-EN ISO/IEC 17025:2005 standards and is accredited by FINAS (the Finnish Accreditation Service; number T024). The dry matter (DM) content was determined by drying samples at 105°C for 16 h, and samples for chemical analyses were dried at 60°C. The ash content was determined using the standard association of analytical communities (AOAC) method (1990, method 942.05). The nitrogen (N) content was determined by the Dumas method (AOAC method 968.06) using a Leco FP 428 nitrogen analyser. The crude protein content was calculated as 6.25 × N content. The concentration of neutral detergent-insoluble fibre (NDF) was determined according to Van Soest et al. (1991) [2] using sodium-sulphite without amylase and presented as ash free. The in vitro cellulase solubility of grass was determined according to Huhtanen et al. (2006) [3]. The crude fat content of the concentrates was measured as the ether extract (method 920.39) after hydrolysis with hydrochloric acid. Determination of the crude fibre content was conducted according to the EEC standard (EEC 92/89, ASN 3802) using the Fibertec 2023 FiberCap system (Foss Tecator AB, Höganäs, Sweden, Application note 3437). The feed energy and protein values were calculated according to Finnish Feed Tables (Luke 2015).

**RNA extraction**

All procedures were conducted at room temperature. Briefly, samples from each ovary were cut into 20-30 mg portions. The tissue samples were disrupted and homogenized in 600 µl of RTL Plus buffer (β-mercaptoethanol was added at 10 µl/ml) using a TissueLyser LT (Qiagen, Hilden, Germany) for 6 min at 50 Hz in the presence of stainless steel beads. After a brief centrifugation at 20,000 x g for 1 min, the homogenized lysate was transferred to an AllPrep DNA Mini spin column and centrifuged 20,000 x g for 30 sec. Proteinase K (50 µl) was added to the flow-through and mixed by pipetting. Subsequently, 200 µl of 100% ethanol was added to the sample and incubated for 10 min. Another 400 µl of 100% ethanol was added and mixed well by repeated pipetting. Each sample was transferred to an RNeasy Mini spin column and centrifuged at 20,000 x g for 15 sec. The total RNA attached to the filter was washed using RPE buffer followed by in-column DNA digestion using DNase I according to the manufacturer's instructions (Qiagen, Hilden, Germany). The filter was washed again using FRN and RPE buffers and 100% ethanol sequentially. Finally, the total RNA was eluted in 60 µl RNase-free water and stored at -80°C until further analysis. The RNA concentration was quantified using a NanoDrop spectrometer set at 260/280 nm (NanoDrop, Wilmington, DE, USA). The RNA quality and integrity were assessed using an Agilent 2100 Bioanalyzer (Agilent Technologies, CA, USA), and all RNA samples had RIN values ≥ 7.8. After adjustment of the RNA concentrations of all pools, cDNA synthesis was performed using random hexamer primers (60 μM), oligo (dT)-18 primers (2.5 μM) and Superscript Reverse Transcriptase II (Roche, Espoo, Finland).

**Supplementary References**

1. Russel A. Body condition scoring of sheep. In Pract. 1984;6:91–3.

2. Van Soest PJ, Robertson JB, Lewis BA, Sniffen CJ, Devries JW, Sniffen CJ, et al. Methods for dietary fiber, neutral detergent fiber, and nonstarch polysaccharides in relation to animal nutrition. J. Dairy Sci. Feedingstuffs Evaluation Unit, 3rd Rep., Rowett Res. Ihstit., Bucksburn, Aberdeen, Scotland; 1991;74:3583–97.

3. Huhtanen P, Nousiainen J, Rine M. Recent developments in forage evauluation with special reference to practical applications. Agric. Food Sci. 2006;15:293–323.

**Supplementary Figures**

**Figure S1**. **(A)** MDS plot based on (IBS) distances calculated using SNP genotype data. The same 31 ewes that were also employed for mRNA and miRNA sequencing were genotyped. **(B)** Principal component analysis (PCA) plot of the top 500 differentially expressed genes.

**Figure S2.** Venn diagram showing the overlap of the top 500 genes among the breeds.


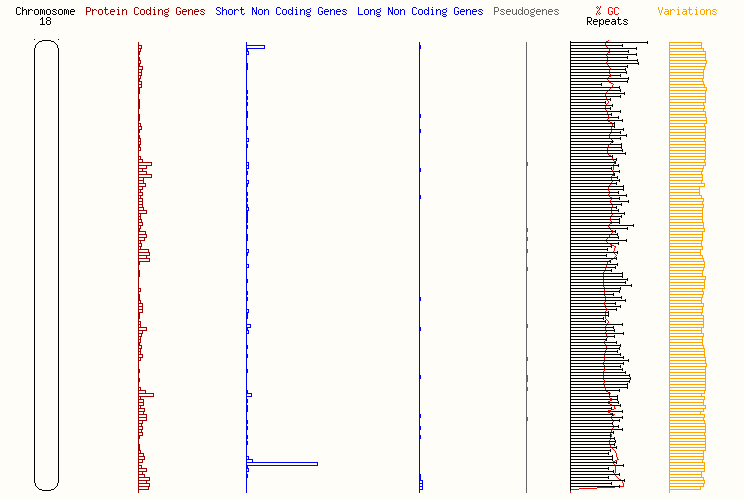


**Figure S3.** Sheep chromosome 8 showing the peak representing the miRNA clusters identified in this study.

**Figure S4.** Pipeline showing the integrated analysis of mRNA and miRNA data.

**Supplementary Tables:**

**Table S1**. Ewes in the trial

|  | |  | |  | |  | | |  | | Litter size at birth | | |
| --- | --- | --- | --- | --- | --- | --- | --- | --- | --- | --- | --- | --- | --- |
| Breed | Treatment | | N^a^ | | Weight^b^ | | Age^c^ | Lambs per year^d^ | | Own | | Parents | Grand parents |
| FI^f^ | Flushing | | 5 | | 72 | | 3.4 | 1.1 | | 2.4 | | 2.4 | 1.9 |
| FI | No flushing | | 4 | | 66 | | 3.9 | 1.0 | | 2.3 | | 2.3 | 1.8 |
| Finnsheep | Flushing | | 6 | | 70 | | 4.7 | 2.4 | | 2.5 | | 3.4 | 2.8 |
| Finnsheep | No flushing | | 5 | | 82 | | 4.8 | 2.1 | | 2.8 | | 2.9 | 3.1 |
| Texel | Flushing | | 6 | | 69 | | 4.0 | 1.3 | | 1.7 | | 1.8 | 1.5 |
| Texel | No flushing | | 5 | | 68 | | 4.7 | 1.0 | | 1.8 | | 1.7 | 1.6 |
| SEM, N=4 | | |  | | 4.4 | | 0.6 | 0.28 | | 0.35 | | 0.24 | 0.16 |
| Breed | | |  | | ns | | ns | *** | | * | | *** | *** |
| Flushing treatment | | |  | | ns | | ns | ns | | ns | | ns | ns |
| Breed*Flushing | | |  | | ns | | ns | ns | | ns | | ns | ns |

^a^Number of observations.

^b^Weight on 7 September 2012.

^c^Age on 3 September 2012.

^d^Number of offspring divided by age.

^e^Number of siblings born at the same time.

^f^Finnsheep x Texel.

SEM = standard error of the mean when n = 4; multiply by 0.894 when n = 5 and by 0.816 when n = 6.

**Table S2**: Compositions of the feed in the feeding trial. Units g/kg DM unless otherwise stated.

|  | DM, | Ash | Crude protein | Crude fat | Crude fibre | OMS, | NDF | ME, | D-value | AAT |
| --- | --- | --- | --- | --- | --- | --- | --- | --- | --- | --- |
|  | g/kg |  |  | g |  | g/kg OM |  | MJ/kg DM |  |  |
| Hay | 927 | 62 | 78 |  |  | 666 | 634 | 9.3 | 610 |  |
| Minerals * | 947 | 796 |  |  |  |  |  |  |  |  |
| Oats | 889 | 35 | 137 | 52 | 100 |  | 281 | 12.0 | 717 | 94 |
| Rape seed meal | 890 | 86 | 337 | 39 | 126 |  | 263 | 11.2 | 690 | 157 |

DM = dry matter.

OMS = cellulase solubility of organic matter.

OM= organic matter.

ME = metabolizable energy.

AAT = amino acids absorbed from the small intestine, calculated according to Finnish feed tables (Luke 2015).

*Mineral feed equivalent to that used during the adaptation period. Composition described in the text.

**Table S3**. Effect of flushing on the body condition scores of the ewes

| Breed | Treatment | β^a^ | Intercept^b^ |
| --- | --- | --- | --- |
| FI | Flushing | 0.00685 | 2.85 |
| FI | No flushing | -0.00020 | 3.21 |
| Finnsheep | Flushing | 0.00563 | 2.86 |
| Finnsheep | No flushing | 0.00000 | 3.20 |
| Texel | Flushing | 0.00638 | 3.28 |
| Texel | No flushing | 0.00480 | 3.25 |
| SEM, N=4 |  | 0.003122 | 0.193 |
| Breed |  | ns | ns |
| Flushing treatment |  | * | ns |
| Breed*Flushing |  | ns | ns |

^a^Linear effect of flushing (number of flushing days) of the BCS.

^b^Intercept predicting the BCS at the start of the flushing.

SEM = standard error of the mean when n = 4; multiply that SEM by 0.894 when n = 5 and by 0.816 when n = 6.

**Table S4**. Summary of the mRNA-seq data. Sample names in the first column also include breed and diet information. For example, TC_11E refers to sample 11E from the Texel breed receiving a control diet, and F1C_19A refers to sample 19A from the F1-cross of Finnsheep and Texel on a control diet. The number of genes (in the last column) include all transcripts with at least 10 reads. One important observation is that the concentration of RNA in the samples does not necessarily affect the number of expressed genes.

| Sample | RNA Concentration (ng/ml) | Raw reads (mil) | Trimmed reads (mil) | Mapping % | # of genes expressed (≥ 10 reads) |
| --- | --- | --- | --- | --- | --- |
| TC_11E | 618 | 79818231 | 79741202 | 81.4 | 14511 |
| TF_13B | 1433 | 43119432 | 43042958 | 78.9 | 14650 |
| F1C_19A | 844 | 25412229 | 25402933 | 76.1 | 14316 |
| FF_21J | 471 | 54836101 | 54763655 | 80.9 | 14642 |
| TF_22A | 1263 | 71278780 | 71197645 | 80.7 | 14520 |
| FF_23C | 1491 | 51688409 | 51652341 | 84 | 14658 |
| F1F_24A | 360 | 52467629 | 52434495 | 82.2 | 14832 |
| TC_26B | 946 | 56771799 | 56750372 | 82.9 | 14778 |
| TC_27A | 639 | 89298427 | 88189642 | 74.7 | 15570 |
| F1C_29A | 628 | 39059761 | 39034977 | 77.3 | 14294 |
| F1F_31A | 737 | 30958957 | 30948379 | 79.7 | 13655 |
| F1F_33A | 1222 | 43467846 | 43444342 | 78.9 | 14335 |
| FF_35C | 464 | 46055562 | 46031154 | 81.5 | 14247 |
| TF_379 | 698 | 61503066 | 61445492 | 82.2 | 15583 |
| F1C_37C5 | 523 | 40293501 | 40274599 | 78.2 | 14131 |
| F1C_37C6 | 523 | 39826175 | 39807599 | 78.5 | 14099 |
| F1C_37C7 | 523 | 38138169 | 38118117 | 78.6 | 14049 |
| FF_39A | 726 | 67991639 | 66429194 | 68.2 | 15197 |
| FF_39E | 1155 | 70869882 | 70651723 | 76.3 | 15153 |
| 3FF_9J | 512 | 69189964 | 68966512 | 77.4 | 15551 |
| TC_3B | 601 | 56095405 | 56047272 | 84.5 | 14607 |
| FC_41B | 1169 | 64219474 | 64138779 | 79.6 | 14925 |
| F1C_4208 | 358 | 41538828 | 41526679 | 78.8 | 14414 |
| FC_42B | 1416 | 54912568 | 54894291 | 81 | 14559 |
| TF_43A | 542 | 28027425 | 28014874 | 79.7 | 13195 |
| TC_44A | 323 | 60882774 | 60647366 | 77.6 | 14863 |
| F1F_4563 | 774 | 74121665 | 73603918 | 77 | 15077 |
| F1F_4590 | 907 | 55576301 | 55444355 | 79.6 | 14367 |
| FC_45A | 628 | 43797554 | 43756222 | 77.3 | 14464 |
| FC_46F | 276 | 71412011 | 71300472 | 78.2 | 15078 |
| TF_4H | 38 | 52222203 | 51842916 | 72 | 14653 |
| TF_4P | 70 | 65849126 | 62956052 | 64.7 | 15002 |
| FF_5B | 517 | 67273296 | 66995814 | 76.8 | 14755 |
| TF_787 | 480 | 76611966 | 76566591 | 83 | 15459 |
| F1F_7G | 573 | 67760274 | 67690256 | 79.1 | 15221 |
| F1F_7M | 800 | 33415970 | 33399015 | 82 | 14547 |
| FF_801 | 893 | 110658646 | 110094637 | 77.3 | 15621 |
| FC_974 | 1285 | 58126166 | 58097220 | 80.6 | 16441 |
| TF_9K | 163 | 77726951 | 77626487 | 79.8 | 15117 |

**Table S5**: Summary of the miRNA-Seq data. Sample names in the first column also contain the breed and diet information, similarly to Supplementary Table S5. For each sample, we removed Illumina adapters and low-quality reads and used clean reads for downstream analysis.

| Sample | Total reads | Trimmed reads | Too short after trimming (< 17 bp) | Aligned reads | Precursor miRNA reads | Mature miRNA reads | Known miRNAs with ≥ 5x coverage |
| --- | --- | --- | --- | --- | --- | --- | --- |
| TC_3B | 9724590 | 9053531 | 513462 | 5812848 | 16593 | 2617621 | 113 |
| TF_4H | 12114329 | 11192524 | 720211 | 6516385 | 14672 | 2201288 | 100 |
| TF_4P | 13296415 | 11993755 | 444601 | 6008932 | 10455 | 1851428 | 95 |
| FF_5B | 12155445 | 11638525 | 352033 | 8661386 | 22994 | 4393324 | 104 |
| F1F_7G | 18983432 | 17780872 | 811921 | 11412441 | 28769 | 6133797 | 117 |
| F1F_7M | 21390359 | 18062662 | 637068 | 13310586 | 29723 | 6080825 | 117 |
| TF_9K | 16996772 | 16460947 | 504014 | 7718860 | 20915 | 2899473 | 106 |
| TC_11E | 30529210 | 27513573 | 647570 | 13290328 | 26337 | 5267637 | 114 |
| TF_13B | 32216419 | 29547386 | 987392 | 26027164 | 76301 | 16496170 | 125 |
| F1C_19A | 24304883 | 23806832 | 732129 | 18096072 | 50421 | 11972992 | 126 |
| FF_21J | 15106670 | 14582523 | 423915 | 9312257 | 24282 | 4697251 | 109 |
| TF_22A | 21478661 | 20912307 | 538050 | 8824783 | 20726 | 3942098 | 116 |
| FF_23C | 23532809 | 21848433 | 750394 | 17106449 | 51447 | 11302630 | 117 |
| F1F_24A | 13062609 | 12787662 | 540423 | 6777536 | 17708 | 2970550 | 107 |
| TC_26B | 13933406 | 13572171 | 645003 | 10051355 | 30528 | 5806188 | 118 |
| TC_27A | 14284551 | 13801317 | 435553 | 8569928 | 23112 | 4240360 | 108 |
| F1C_29A | 12396579 | 12103821 | 463044 | 8055781 | 21545 | 4557816 | 107 |
| F1F_31A | 9589700 | 8746030 | 756753 | 5826436 | 15389 | 2426799 | 102 |
| F1F_33A | 10085787 | 9243930 | 684481 | 6008832 | 17643 | 2790289 | 117 |
| FF_35C | 16341570 | 15836744 | 486470 | 8939463 | 24675 | 4564617 | 110 |
| F1C_37 | 10011902 | 9182606 | 918176 | 5699317 | 14872 | 2649020 | 110 |
| FF_39A | 14813422 | 13846593 | 650800 | 9282185 | 22322 | 4620204 | 101 |
| FF_39E | 19983619 | 18513920 | 677717 | 15247192 | 42951 | 9515528 | 120 |
| FF_39J | 16842777 | 15716371 | 719926 | 11124735 | 27402 | 6024528 | 111 |
| FC_42B | 11650562 | 11414663 | 429995 | 8699028 | 22724 | 5453135 | 108 |
| TF_43A | 9784668 | 9567944 | 404665 | 5331197 | 13398 | 3179804 | 121 |
| TC_44A | 26760319 | 25933176 | 639918 | 13855132 | 35425 | 6693493 | 117 |
| FC_45A | 15330459 | 15087284 | 499004 | 10859031 | 25155 | 7132948 | 114 |
| FC_46F | 20283305 | 19026126 | 566460 | 12028230 | 33171 | 6347428 | 109 |
| TF_379 | 19750740 | 19113362 | 501826 | 13694829 | 32824 | 8130227 | 116 |
| FC_41B | 33135636 | 28787226 | 750618 | 21365726 | 47990 | 11277913 | 122 |
| TF_787 | 14029588 | 12936792 | 549759 | 10857201 | 32270 | 6345173 | 111 |
| FF_801 | 12000512 | 11001410 | 480500 | 8605301 | 27292 | 5011220 | 108 |
| FC_974 | 21048622 | 19864374 | 780670 | 15696674 | 38965 | 10565620 | 123 |
| F1C_4208 | 10612480 | 10349357 | 608709 | 6397042 | 17845 | 3135242 | 106 |
| F1F_4563 | 21247067 | 18931498 | 579745 | 10233579 | 24850 | 4612063 | 112 |
| F1F_4590 | 17497974 | 15985537 | 998505 | 11268509 | 27800 | 6006763 | 123 |
